# mHealth Interventions to Promote Anti-Retroviral Adherence in HIV: Narrative Review

**DOI:** 10.2196/14739

**Published:** 2020-08-28

**Authors:** Stephen B Lee, Joanne Valerius

**Affiliations:** 1 Department of Medicine, Division of Infectious Diseases University of Saskatchewan College of Medicine Regina, SK Canada; 2 Department of Medical Informatics and Clinical Epidemiology Oregon Health and Science University Portland, OR United States

**Keywords:** mHealth, HIV, antiretroviral, adherence, mobile phone

## Abstract

**Background:**

Antiretrovirals (ARVs) are key in the management of HIV. Although no cure exists, ARVs help patients live healthy lives and prevent transmission to others. Adherence to complex regimens is paramount to outcomes and in avoiding the emergence of drug-resistant viruses. The goal of therapy is to reach an undetectable viral load. However, adherence is a common problem, stemming from issues such as mental health, chaotic home situations, and busy work schedules. Mobile health (mHealth) represents a new approach in improving medication adherence, and multiple studies have been performed in this area.

**Objective:**

This study aims to review the current implementation of mHealth in the management of HIV among different groups of patients.

**Methods:**

We used PubMed, Academic Search Elite, and 1 journal database with various search terms to review the current implementation of mHealth in HIV care.

**Results:**

Titles and abstracts were screened, and 61 papers were identified and fully reviewed. The literature was divided into lower- and higher-income nations, as defined by the United Nations. A total of 20 studies with quantitative results were identified, with 10 being text- and SMS-based interventions (the majority of these being in lower-income countries) and 8 being smartphone-based apps (primarily in higher-income countries). The majority of these studies determined whether there was an effect on adherence or biochemical parameters (viral load and CD4 count). Various qualitative studies have also been conducted, and many have focused on determining the specific design of interventions that were successful (frequency of messaging, types of messages, etc) as well as priorities for patients with regard to mHealth interventions.

**Conclusions:**

There seems to be a role of mHealth in the management of HIV in lower-income nations; however, the optimal design of an intervention needs to be delineated. In higher-income countries, where the 2 significant risk factors were injection drugs and men who have sex with men, the benefit was less clear, and more research is needed.

## Introduction

### Background

HIV remains to be a significant public health concern, causing an estimated 770,000 deaths worldwide, with significant mortality from poorly managed disease, even in developed nations [1,2]. Particularly in the developing world, there is significant morbidity with 79.1% of HIV prevalence centered in Africa (25,700,000 people) and Southeast Asia (3,500,000 people) [3,4].

The burden of this disease is not only health related but also impacts economics and development. A large proportion of deaths from HIV occurs in young and middle-aged adults; the same group of people who raise children, are caretakers, work, and teach future generations. Especially in lower-income countries, families who have had a death from HIV cope by pulling children out of school, reducing food intake, or using up agricultural resources prematurely [5]. In addition, although first-line antiretrovirals (ARVs) cost US $80 per year, the cost of second- and third-line regiments (used with drug resistance) may be as high as US $2200 per year [6].

Despite all these bleak consequences, studies have shown that with adherence to ARV therapy, a patient aged 20 years could expect an additional 43.3 years of life [7]. In contrast, those who are not adherent and develop AIDS have a median survival of 12.1 months [8]. Adherence to ARVs is key to prolonging life, improving immune function and quality of life, and preventing transmission [9-14].

Without proper adherence, there is a risk of disease progression or drug resistance. Despite increasing access to ARVs worldwide, adherence is far from perfect [15,16]. One meta-analysis reported adherence at 62%, and studies on subgroups, such as illicit drug users, are as low as 27% [17,18].

Factors such as psychiatric disorders, cognitive impairment, social stigma, substance abuse, and volatile housing can prevent optimal adherence [19,20]. Burch et al [21] found clear associations between markers of the social determinants of health and HIV outcomes. Rachlis et al [22] found that substance abuse and unemployment were associated with suboptimal care. The risk of transmission and resistance make it crucial to develop methods for improving adherence to medications. Indeed, international organizations have called for strategies to improve adherence to medication and its importance [23-25].

Consumer informatics, particularly mobile health (mHealth), has shown promise in improving ARV adherence. Indeed, research has shown that patients living with HIV are interested in apps to support HIV self-management, and much past research has been done to support disease management [26,27].

Given the widespread adoption of cellphones (95% of the population) and smartphones (77%) in different parts of the world, mHealth is becoming an important tool to improve care [28]. Furthermore, in North America, access to mobile data and the internet ranges close to 80%, and with expanding mobile computing capabilities, this represents further opportunities [29].

### Objectives

We believe that the major benefits that mHealth could offer would be helping with medication and appointment adherence, improving HIV education, and increasing engagement in care. Some studies have shown promise in improving viral suppression and CD4 count. However, there are existing gaps in current reviews [30]. First, as technology is a rapidly growing field, constantly updated work is needed to summarize developments. Second, and most importantly, people who live with HIV are a diverse group of patients with a variety of challenges, including living in developing countries with poor health infrastructure and having comorbid substance use issues [31-34]. Each of these patient populations has unique characteristics and concerns that may influence care. For instance, access to the internet and smartphones, health care, income, and psychiatric comorbidities could be vastly different in various groups. The majority of existing reviews homogenize and summarize the existing literature as a whole and do not consider the differences in patient populations [35-39]. An intervention may have varying levels of success in each group and likely needs to be tailored to fit unique needs. For instance, a smartphone app for disease education may be successful in college-educated, smartphone-owning patients; however, it may have a vastly different effect on a patient in the developing world without a smartphone or an advanced education. The underlying differences in patient groups need to be considered, and our review groups studies into subgroups and discusses the implications of such differences.

## Methods

### Search Strategy

Two separate databases with peer-reviewed, reputable articles were used, PubMed and Academic Search Elite. All relevant articles reviewed were within the time period of 2010 to 2019.

For PubMed, we used the following search terms: “mHealth AND HIV AND medication adherence,” “app AND HIV AND adherence,” “smartphone AND app AND HIV AND anti-retroviral adherence,” and “mobile phone AND anti-retroviral adherence AND HIV.” On reviewing the literature using these search terms, we added the search terms “WelTel” and “CAMPS and retroviral” to capture relevant studies found in the references of the articles retrieved from the initial search.

We used the search terms “mHealth AND HIV AND medication adherence,” “mobile phone AND anti-retroviral adherence AND HIV,” and “app AND HIV AND adherence” for the Academic Search Elite database search.

Finally, we used the *Journal of Medical Internet Research* database, a group of journals that focuses on informatics implementations in health care, to identify articles listed under “mHealth for Treatment Adherence.”

### Inclusion and Exclusion Criteria

A total of 386 search results were screened using the title or abstracts for relevance, and 61 articles were fully reviewed and are discussed in this study.

Articles were included if they were experimental in nature (encompassing qualitative or quantitative studies as well as randomized controlled trials [RCTs] or other studies). Study designs included RCTs, case studies, cohort studies, cross-sectional studies, and qualitative studies (focus groups, interviews, observations, and surveys). No age restrictions were set, although the majority of the literature focused on adults and studies were required to have all participants diagnosed with HIV. A significant number of articles were excluded (273/325, 84%). Reasons for exclusion were irrelevance to the topic discussed, inclusion of patients not diagnosed with HIV, articles written in a language other than English, reviews rather than original research on this topic, categorization as nonexperimental irrespective of whether qualitative or quantitative (for instance, a commentary of mHealth), or if they were protocols for experiments.

We defined mHealth in this study as any intervention that involved the use of mobile phones, smartphones, or other wireless devices (such as smart pill bottles). Although no formal definition of mHealth exists, this description is in agreement with the World Health Organization [40]. Pilot trials found on literature review were included in our review.

## Results

### Overview

A total of 61 papers were identified from the period of 2010 to 2019. In accordance with the mHealth categories previously described by the United Nations, we categorized apps according to type and found that these apps were focused on either treatment support (reminders) or education [41]. In addition, we categorized apps as either push or pull and as one-way versus dual communication. Interventions that were primarily reminders tended to use push, whereas those that were education used pull (Table 1).

As factors such as technology accessibility, socioeconomic background, and internet or cellular connectivity vary based on geographical location, the authors chose to separate the discussion into studies performed in lower- and higher-income countries. Studies were divided into categories (*upper middle and upper* vs *lower middle and low*) as per the definition by the United Nations [42].

The risk factor for HIV acquisition in upper middle- and upper-income countries was primarily men who have sex with men (MSM) or intravenous drug users (IVDU). The majority of these studies were performed in North America (Canada and the United States).

For lower middle- and low-income countries, the majority of these studies were based on the African continent, with some conducted in Asia.

Interventions were text or SMS based, voice message based, or smartphone apps. All work in the lower-income countries consisted of simple text or SMS or voice interventions (7/7, 100% of quantitative studies) [43-49]. In contrast, in higher-income countries, more of the work included advanced smartphone apps (8/13, 62%; Table 1) [50-58]. Although we have summarized the quantitative studies on efficacy in Table 1, other studies discussed the optimal designs or considerations in intervention development and are reviewed in the following sections.

On an evaluation, using the Oxford Centre for Evidence-Based Medicine’s Levels of Evidence, there was a large amount of high-level evidence in developing countries, with many RCTs [59]. On the basis of this, we believe that conclusions regarding efficacy are possible. There is also a high level of evidence (with a large number of RCTs) in higher-income countries; however, there remain some limitations to those studies (Table 1). The authors believe that further work may need to be performed in higher-income countries.

**Table 1:** Quantitative studies on the effects of mobile health and ubiquitous Health interventions in HIV management and antiretroviral adherence.

Modes of intervention			Patient population	Designs	Effects	Push (central) vs pull (client) vs other	One-way vs dual communication	App type
**Text message/SMS based (n=10)**								
	**Resource-limited settings (n=5)**							
		Lester et al [43]; WelTel Kenya1	Low income, primarily urban population in Nairobi who were: Starting ARVs^a^ for the first time >18 years old Daily access to mobile phone. 538 randomized: 273 SMS and 265 control in primary analysis	Individually randomized multisite to SMS or control; intervention was weekly SMS with response required within 48 hours; primarily zidovudine or stavudine+lamivudine+efavirenz or nevirapine	Improved rate of viral suppression (57% vs 48%, *P*=.04); improved self-reported adherence (62% vs 50%; *P*=.006)	Push	Dual	Diagnostic and treatment support (reminder)
		Pop-Eleches et al [44]	Rural government clinic in Kenya. Patients were >18 years old Patients were started ARV 3 months earlier	720 patients randomized to short daily (70), long daily (72), short weekly (73), and long weekly (74) messages. Data gathered from MEMS^b^ caps	Improved adherence of >90% (53% vs 40%; *P*=.03); decreased lapses of >48 hours (in the weekly message subgroup; 81% vs 90%; *P*=.03)	Push	One way	Diagnostic and treatment support (reminder)
		Maduka et al [45]; SMS and counseling	Tertiary care Nigerian hospital, patients who had the following: ARVs for ≥3 months History of nonadherence (<95%) Access to mobile SMS	104 randomized to intervention of monthly adherence counseling and twice weekly ARV (52) or control with no SMS or counseling (52)	Improved self-reported adherence (76.9% vs 55.8%; *P*=.02); improved CD4 (193-575 cells/mL vs 131-361.5 cells/mL; *P*=.007)	Push	Dual	Diagnostic and treatment support (reminder)
		Haberer et al [46]	Rural Southwestern Uganda. Patients were >18 years old Patients were initiating ARVs Patients had an own operational cell Patients had 1-2 social supporters Patients were close to hospital	63 patients who were randomized to scheduled SMS and RTAM^c^ (21), triggered SMS from RTAM (20), or RTAM only (21); the scheduled SMS group was daily for 1 month and weekly for 2 months, followed by reminders triggered by late or missed doses. SMS was also sent to *social supporters* if there was no signal >48 hours; triggered SMS was sent only in response to late or missed doses	Improved adherence (91% vs 79% vs 79%; *P*=.04); decreased lapses (7 vs 11 vs 11 for 48 hours and 1 vs 3 vs 4 at 96 hours; *P*=.02); no significant differences in VL^d^ suppression	Other (one arm was *push* with scheduled messages, another arm was triggered by smart pill bottle)	One way	Diagnostic and treatment support (reminder)
		Mbuagbaw et al [47]	Hospital in Yaounde, Cameroon with patients who: Are >21 years old Owned mobile phone, could read, and text Had >1 month of ARV	200 patients randomized to weekly SMS developed from focus groups that were varied, contemporary (eg, Season’s Greetings), with a call back number (101) vs usual care (99)	No significant difference in adherence	Push	Dual	Diagnostic and treatment support (reminder)
	**Higher-income countries (n=5)**							
		Guo et al [60]	Large metropolitan South Chinese hospital. Patients were >18 years old Patients had >1 month of ARVs Patients were able to read or write	62 primarily nonheterosexual males who were randomized to weekly SMS and reminders for ARVs and exercise with WeChat educational materials sent 3 times per month (31) or control (31)	No significant difference in CD4 counts or missed medications	Push	Dual	Diagnostic and treatment support (reminder)
		Moore et al [61]; SMS for adherence and drug use, iTAB	Pilot at UCSD^e^ whose patients: Were >18 years old Had DSM-IV-TR^f^ diagnosis of methamphetamine abuse or dependence or self-reported use within 45 days Were willing to participate in study components	75 randomized to iTAB, which were daily personalized texts built from focus groups and focused on responsibility to others, self-esteem, nonadherence risks, harm reduction, reminders, spirituality, celebration of health, and disease control (50) vs control (25). Also assessed methamphetamine use with daily texts	No significant difference in adherence (measured by MEMS caps); fewer methamphetamine use days (*P*=.05)	Push	Dual	Diagnostic and treatment support (reminder)
		Ingersoll et al [51]; SMS including adherence, mood, and substance use	Pilot study from 2 outpatient clinics in Virginia serving mainly nonurban and rural, primarily male and white, patients who: Were >18 years old Had an active prescription for ARVs Had <95% adherence in the past 2 weeks Consumed illicit drugs or had a risky level of drinking within 30 days Had good command of English	63 patients randomized to daily texts on medications, twice daily mood texts, and daily substance use texts eliciting responses from patients (33) or usual care (30)	No significant difference in proportion of missed visits (*P*=.12); no difference in substance use days; improved adherence (66%-85% vs 62%-71%; *P*=.04)	Push	Dual	Diagnostic and treatment support (reminder)
		King et al [62]	Repeated measures study where patients recruited from a clinic for women and families living with HIV in Vancouver, British Columbia, who: Attended a clinic for at least 1 year Had CD4<500 cells/mm^3^ Were detectable VL 1 year prior Were ≥14 Had a high risk for disengagement	85 enrolled with 5 lost and administered intervention, which was modeled after WelTelKenya1 with SMS or texts every Monday asking “How are you?” requiring a response from patients within 1 day. If no response, this was followed-up by further texts and calls	Mean VL decreased from 1098 copies/mL to 439 copies/mL (*P*=.004); adherence to ARV (OR 1.14; *P*<.001); decreased appointment adherence (OR 0.81; *P*=.03)	Push	Dual	Diagnostic and treatment support (reminder)
		Rana et al [63]	Patients recruited from Rhode Island clinic providing HIV services, primarily white, and MSM^i^ as a risk factor with patients who: Were ≥18 years Had a cell phone capable of texting Were newly engaged in care within 1 year or re-engaging after a lapse of >1 year or at risk for nonadherence	32 patients enrolled, with 20 completing study (exclusions were due to death, incarceration, transferal of care, or no response) given a daily bidirectional texting intervention	Improved VL suppression (*P*=.002)	Push	Dual	Diagnostic and treatment support (reminder)
**Smartphone or computer apps (n=8)**								
	**Higher-income countries (n=8)**							
		Venter et al [54]; lab results, appointment reminders, disease information	Multisite RCT^h^ in the inner city of Johannesburg, South Africa (1 community health center, 3 clinics, 1 tertiary care hospital) with patients who: Were >18 years old Read English or Zulu Were residents of the area	353 randomized to SmartLink, which provided appointment reminders, information about lab tests, ARV adherence and HIV info, and CD4 and VL results (181) as well as control (172)	No significant difference in adherence; improved linkage to care in the 18- to 30-year-old subgroup (20% increase, *P*=.02)	Push	One way	Diagnostic and treatment support (reminder) and education and awareness
		Himelhoch t al [50]	Pilot study from a Baltimore urban outpatient HIV clinic whose patients: Were aged 18-64 years Were patients of the clinic’s adherence program. The adherence counselor determined difficulty in adherence. Had self-reported history of drug or alcohol use Carried mobile phones	30 patients randomized to the Heart2HAART app giving medication reminders; information regarding adherence; ecological momentary for side effects, depression, and cravings for drug use and tailored education; recommendation; and encouragement (20) vs control of smartphone only (10)	No significant difference in adherence	Push	One way	Diagnostic and treatment support (reminder) and education and awareness
		Dillingham et al [52]	Study from a clinic in Virginia whose patients were: New (within 90 days) HIV diagnosis, returning to care after lapse, or considered elevated risk of nonadherence Fourth grade reading level or better	77 enrolled in study primarily male, slightly less than half MSM given PositiveLinks iteratively designed user-centered app with tailored resources, queries of mood, stress, adherence; appointment reminders, and community forum	Improved retention in care at 12 months (*P*<.001); improved constant visits (*P*<.001); improved CD4 count (*P*<.001); improved VL suppression (*P*≤.001)	Pull	Dual	Diagnostic and treatment support (reminder) and education and awareness
		Horvath et al [53]	Pilot study recruited from ads on Grindr from Miami, Orlando, Washington DC, Charlotte, Houston, and New Orleans as well as flyers or cards from organizations and clinics serving the targeted population. Patients were: Male and MSM within 5 years A US resident Currently taking ARVs Reporting suboptimal adherence The use of illicit stimulants (eg, methamphetamines, cocaine, ecstasy, amphetamines) within 6 months Owning an iPhone or Android phone	90 patients randomized to APP+ for MSM who use stimulants, giving informational material, a playable storyline of a fictional HIV+ character, and a tool to track personal adherence (45) vs control (45)	Temporary significant improvement in self-reported adherence (*P*=.04); no significant difference in substance use	Pull	One way	Education and awareness
		Dworkin et al [55]	Patients in Chicago who were: Aged 18-34 years old On ARVs for >3 months Owning an Android smartphone Scheduled for a blood draw or visit during 3 months after baseline assessment African American MSM	43 patients enrolled, with 11 lost to follow-up, given avatar conversational agent intervention on mobile phones	Improved adherence (via pill count adherence >80%; *P*=.05)	Pull	One way	Education and awareness
		Whiteley et al [56]	Patients recruited from the greater Jackson, MS area who were: 14-26 years of age Receiving or starting ARVs Aware of status Detectable VL within 1 month of screening Speaking English Able to consent	66 patients who were primarily male, black, and nonheterosexual enrolled, with 5 patients lost receiving BattleViro mobile game designed from qualitative user feedback that helped ARV adherence, viral load, and other knowledge regarding HIV	Greater decrease in VL (0.96 log greater decrease in intervention; *P*=.04); improved adherence (71% vs 48%; *P*=.05)	Pull	One way	Education and awareness
		Perera et al [57]	Patients who were primarily male and homosexual, recruited from the Auckland City Hospital Infectious Diseases Clinic and local HIV support organization who were: On ARVs for at least 6 months Using Android smartphones	28 randomized to augmented apps, including graphical representations of ARV concentrations and simulation of immune activity (17) vs normal apps with medication clock only (11)	Increased self-reported adherence via MARS^i^ score (48.93 vs 47.09; *P*=.03); lower VL at 3 months (1.30 log10 vs 1.70 log10; *P*=.02)	Pull	One way	Education and awareness
		Ownby et al [58]	Recruitment of patients in Broward County, FL with a large proportion of MSM^g^	124 participants recruited in educational intervention, with a final 109 patients analyzed for results	Improved adherence in subgroups with lower baseline adherence rates (in <85% adherence, *P*=.04; in <80% adherence *P*=.09)	Pull	One way	Education and awareness
**Voice calls (n=2)**								
	**Resource-limited settings (n=2)**							
		Shet et al [48]; HIVIND**,** interactive automated voice all and reminder pictorial messages	1 ambulatory and 1 private HIV clinics in India whose patients: Were aged 18-60 years Were ARV naïve Had first-line ARV: (zidovudine or stavudine or tenofovir+lamivudine+nevirapine or efavirenz)	631 educated patients randomized to motivational call with inquiry of adherence requiring response and pictorial SMS (315) or control (316)	No significant difference in viral load; no significant difference in adherence	Push	Dual	Diagnostic and treatment support (reminder)
		Rodrigues et al [49]	Bangalore, South Indian tertiary, nonprofit private facilities outpatients who: Had access to mobile phones Had >1 month on ARVs Had first-line regimens (zidovudine or stavudine+lamivudine+nevirapine or efavirenz)	Quasi experimental, where 150 primarily male patients were all given automated interactive voice response call and noninteractive neutral picture SMS every week for 6 months and studied at the end of the intervention	Improved adherence (85%-91% during intervention and 6 months after, *P*=.02)	Push	Dual	Diagnostic and treatment support (reminder)

^a^ARV: antiretroviral.

^b^MEMS: medication event monitoring system

^c^RTAM: real-time adherence monitoring.

^d^VL: viral load.

^e^UCSD: University of California at San Diego.

^f^DSM-IV-TR: Diagnostic and Statistical Manual of Mental Disorders – IV – Text Revision.

^g^MSM: men who have sex with men.

^h^RCT: randomized controlled trial.

^i^MARS: medication adherence report scale.

### Lower-Income Countries Group

Given the rising rates of cellular access in the developing world, mHealth represents a significant opportunity to improve care [64].

A variety of studies have been conducted on mHealth interventions in the developing world, with a wide range of study methodologies. The quantitative experimental designs (including cohort studies and RCTs) are summarized in Table 1 for ease of comparison. Seven studies with quantitative data were available, with the majority being text- or SMS-based modalities (5 studies) and a minority being voice call based (2 studies) [43-49].

Other studies investigated user acceptability and surveyed patient concerns regarding the use of mHealth. A pilot study in rural Uganda by Musiimenta et al [65] enrolled 63 patients and randomized them to a scheduled SMS, triggered SMS (by missed or delayed doses), or no SMS. The feedback received was positive, citing usefulness for motivation, reminders, and social support. However, confidentiality, shared phones, ability to use the phone, and availability of electricity were concerns [65]. Similarly, an explorative study by Lepere et al [66] demonstrated that mHealth was acceptable among people who live with HIV in Cote d’Ivoire, Togo, and Burkina Faso and advocated for its use. Mbuagbaw et al [67] also demonstrated, in a qualitative arm of the CAMPS trial, that these interventions were acceptable, although content, timing, technical challenges, and privacy need to be considered.

### Higher-Income Countries Group

Countries classified as high or high middle income were analyzed. The primary risk factors in these studies were either IVDU or MSM, in agreement with the previous literature [68,69].

Quantitative experimental studies are described in Table 1 [50-58,60-63,70]. In addition, a number of nonquantitative or nonexperimental studies were conducted to investigate the nature of optimal interventions. These are summarized in Table 2 [71-84].

Different studies have investigated aspects of mHealth apps other than efficacy. WelTel was studied in Vancouver, British Columbia, on patients involved with substance abuse using both quantitative and qualitative methodologies [62,84]. One such study was done by Campbell et al [85], who studied WelTelBC1 from an economic perspective on 85 viral load–positive vulnerable patients (defined as substance use and other psychosocial factors), finding that the cost of caring for a highly vulnerable patient was US $347.74, whereas the overall median cost was US $36.72.

Multiple quantitative studies investigated the acceptability of mHealth interventions. In Seattle, 224 patients with HIV were randomized to receive two-way pagers with personalized adherence messages. Of these patients, 55% identified as gay or lesbian, and the majority were white males. Participants were surveyed for the usefulness of the pager and asked whether they adhered to their medications; 73% reported liking the pager, 51% began finding messages annoying, and 48% believed that the pager did not improve their adherence. The overall response rate was only 42.8% but declined over 3 months. The self-reported pager adherence was 90.8% (SD 33%) compared with the medication event monitoring system showing 53.6% (SD 37%) adherence [86].

Similarly, a Peruvian study by Krishnan et al [87] with 359 transgender women or MSM patients asked about mHealth preferences using a 5-point Likert scale. They found an overall positive uptake, with a mean of 3.21 for technological medication reminders and 3.56 for anonymous internet chats to discuss HIV issues.

Other studies, using focus groups and other quantitative study methods, were used to determine how best to deliver mHealth interventions. For instance, Krishnan et al [87] also found that daily messages were preferred in terms of frequency within their study population. In Bangkok, Anand et al [88] interviewed 16 MSM and 2 transgender women. They gathered information on preferences and how to address needs with electronic health. The group found that 39% (7/18) of patients preferred instant messaging and 11% (2/18) preferred phone calls. Of these, 39% (7/18) wanted a private website chat room and 11% (2/18) preferred Skype (Skype Communications) video chats. All patients desired personalized reminder messages; 50% (9/18) wanted reminders on an instant message app, 17% (3/18) preferred a stand-alone app, and 33% (6/18) wanted it as an SMS. The theme that emerged was web based, accessible, and reliable disease information [88].

Work performed in higher-income countries also had diversity in intervention type. For instance, Skrajner et al [89] enrolled a patient with cognitive impairment in a video-conferenced, cognitive-psychosocial program that improved the pill count adherence rate from 75% to 97.9% in 1 month (80% in 2-3 months). Similarly, the weCare team, which has done non-HIV work on social media, is currently developing a tailored Facebook, text, and social and sexual networking app–based intervention to help engage care [90]. Hwayoung et al [91] combined an electronic pill bottle, fitness tracker, and phone alerts and found that the pill bottle encouraged adherence and self-management of medications. However, they mentioned that the smart pill bottle was too small to fit all the pills, although they liked how it was easy to open and discrete [91].

Finally, Schnall et al [92] created the mVIP app, which attempted to provide self-care strategies for self-management of different HIV-related symptoms. They found improvements in a variety of symptoms, including anxiety (*P*=.001), depression (*P*=.001), neuropathy (*P*=.002), fevers or chills or sweats (*P*=.04), and weight loss (*P*=.02). Results for adherence were mixed, showing improvement only when measuring it via the center for adherence support evaluation adherence index [92].

**Table 2:** Qualitative studies on the priorities and important themes in mobile health interventions.

Study	Designs	Themes reported by patients
Senn et al [71]	22 African American MSM^a^ with HIV in Rochester completed surveys and qualitative interviews	Importance of social support Convenience Anonymous and confidential
LeGrand et al [72,73]	EPIC Allies (University of North Carolina, Duke University) undergoing study now. Game-like app that uses game mechanics and social networking to improve HIV care. Development study looked at ARV^b^ adherence needs, preferences, and usability testing in young MSM and transgendered women who have sex with men	Information about side effects Discrete medication reminders Interactive Engaging Social Informational Customizable
Dworkin et al [74]	5 different focus groups composed of African American MSM with HIV in Chicago iteratively designed a talking avatar app providing information about disease, improved adherence, and helped with appointment attendance	Positive impression Importance of confidentiality Motivational over negative messages Customizable
Castel et al [75]	Focus groups and surveys on patients with HIV aged between 13 and 24 year (no delineation as to acquisition) for 3 different game prototypes that were linked with Wisepill dispensers	Game was feasible and acceptable Customizable, challenging, and user-friendly games
Morano et al [76,77]	Primarily, 132 African American males enrolled to the Care4Today app that helped with medication management and appointments and tracked health, wellness, and goals	Higher education levels, staff support, and possession of smart phone predicted use of app 70.2% of patients interested in medication reminders
Cook et al [78]	37 patients in Colorado studied to determine if messages matched to psychological states were useful. Patients were primarily ethnic minorities and nonheterosexual	Changing messages improved adherence (potentially indicating importance of novelty)
Olalla et al [79]	Spanish study on 30 patients (15 randomized to app group) who were >60 years old. The app offered medical news, reports about HIV, and an anonymous chat between patients	No clinical or analytical parameter changes (likely due to short intervention period and small sample) Patients enjoyed the social aspect of the app Analysis of the chat logs showed interested in general health over HIV specifically Most patients seemed to have positive emotions and often mentioned happiness
Westergaard et al [80]	Recruited patients with HIV who were absent from appointments, had substance use, and had an unsuppressed viral load. Investigated acceptability and uptake of a 2-part intervention that included a smartphone app (delivering tailored interventions and communication) and a peer navigator (psychosocial and logistical support)	The participants universally commented on the app’s usefulness as medication and appointment reminders Some requested alternate apps at the end of the trial
Przybyla et al [81]	Used app for adult patients with HIV who had been on ARVs for >3 months with history of alcohol and a history of at least one recent day of nonadherence. Evaluated feasibility and acceptability of the app	Found high report completion rates demonstrating feasibility and acceptability of the app Challenges include those with limited smartphone experience
Horvath et al [82]	Focus groups for stimulant using MSM with HIV (San Francisco, Minneapolis) and explored app components that were important for continued use and engagement	Important features included: low cost, customizable, integrate well into their lives, be engaging, credible, private, and provide appointment and medication reminders
Rosen et al [83]	Focus groups of patients with HIV with a history of substance abuse using the *iHAART* app (which used visual representations of adherence, VL^c^, and CD4)	Need a balance of requested and provided information Emotions provoked by the app can affect adherence
Smillie et al [84]	Pilot WelTel study in Vancouver, British Columbia, on patients with substance abuse using a qualitative methodology of semistructured interviews	Participants thought app was more useful as means of accessing psychosocial support and health care providers rather than as a reminder or source of information

^a^MSM: men who have sex with men.

^b^ARV: antiretroviral.

^c^VL: viral load.

## Discussion

### Lower-Income Countries Group

Many studies have been performed using mHealth to improve adherence, with most in sub-Saharan Africa. Some of the studies were rigorous, large RCTs, such as Kenya WelTel [43]. There were some limitations in other studies where mHealth was not studied independently. For instance, in the study by Maduka et al [45], the effects of adherence counseling and SMS messages were not separated, and in the trial by Haberer et al [46], the role of social supporters was unclear. However, most of the studies indicate an improvement in adherence and VL (Table 1) [43-46,48,49]. Furthermore, it seems that mHealth represents a cost-effective intervention compared with the alternatives, with 1 study showing that a nurse could manage 1000 patients per week [43].

Although most studies found benefits, there is a gap in the literature regarding the design of a successful intervention. It was found that daily messages were not optimal, habituation likely being a significant factor [43-47]. Pop-Eleches et al [44] found that weekly interventions were more effective than daily interventions and long versus short messages made no difference, potentially reflecting that the messages were used more as reminders.

Most other successful interventions agreed with tapering schedules, twice weekly messaging in Nigeria, weekly WelTel Kenya1 messages, and the weekly South Indian study [43,45,46,49]. Another variable was message interactivity and content. On the basis of the existing literature, it is unclear if the interactivity of messages is important. In terms of content, Pop-Eleches et al [44] showed that a motivational message “This is your reminder. Be strong and courageous, we care about you” was not better than a generic impersonal message. These findings potentially indicate that an interactive or customized message does not negate the effects of habituation. Further work is necessary to determine the exact type of message that generates the maximal benefit.

Cultural contexts likely play a role in feasibility, and caution should be exercised when extrapolating results to different locations. Different views on health care, HIV stigma, baseline demographics, and cultural or religious views could be important when designing an intervention. The HIVIND study illustrates this, with high baseline adherence further boosted by the Hawthorne effect [48].

These studies collectively show a role for mHealth in improving HIV care in low-income or lower middle-income nations; however, we still need to delineate the best design. A less-than-daily message frequency seems to be optimal; however, interactivity and content need to be considered. Geographical and cultural differences may also affect efficacy.

### Higher-Income Countries Group

The major risk factor for HIV in higher-income countries was either IVDU or MSM [93-98]. Although in higher-income countries different factors were identified in HIV management compared with lower-income countries, optimizing adherence is still the key.

In those with IVDU as a risk factor, compliance is important as it has implications for disease transmission. Finding meaningful interventions to improve adherence is crucial as there are higher rates of drug use in patients with HIV+, and these patients often face barriers to adherence [20,99].

However, there are a series of unique challenges for these patients that have implications in implementation and interpretation of studies. An inherent difficulty in studies performed on patients with a history of substance misuse is their heterogeneity, with a variety of substances used, including alcohol, marijuana, cocaine, amphetamines, and opioids. Prolonged use of drugs such as methamphetamines can cause psychotic episodes, resulting in more chaotic lives than other addictive substances [100,101]. Opioids have substitution agents that represent an opportunity for frequent, recurrent health care interaction in dispensing [102]. An intervention efficacious in one group may not be efficacious in another.

In addition, a variety of barriers exist that confound the ability of mHealth interventions to help with adherence. These include unstable financial and housing situations as well as psychiatric comorbidities [103]. Some of these barriers may limit the ability for any intervention to be beneficial without first addressing them.

The majority of the interventions studied in patients with substance misuse were text or SMS based, whereas those focusing on populations with MSM as the major risk factor had more advanced smartphone apps (Table 1) [50-58,60-63]. A potential explanation is the financial resources required for a smartphone and an accompanying plan, which supports the need for stable financial situations before a successful intervention.

Overall, across risk groups in higher-income countries, there seemed to be a theme of advanced mHealth interventions (using apps, social media, and game-like interventions)**.** Examples in our review included EPIC Allies, the talking avatar, and the weCare interventions that contrasted mainly to call and text-related interventions in lower-income countries [72-74,90]. This could potentially be explained by the availability of smartphones and internet connectivity in higher-income countries [28,29].

Regardless of risk factors, a limitation of many of these individual studies was the small sample size. For instance, the iTAB study only showed an effect on methamphetamine use, but as this factor could contribute to nonadherence, a larger sample size may have had an effect on adherence [61].

In addition, studies outside North America should be interpreted with caution if implemented in North America, as many different considerations exist. For instance, different regions of the world preferentially use different messaging apps [104]. These may have different functions and capabilities. Certainly, further work is required [105,106].

Design-related studies indicated the themes that interventions should be customizable, provide disease information, and maintain confidentiality [71-84]. A reminder intervention seems to be acceptable in the patients studied [76,77,80,82]. The role of applications as a conduit for health care support was also emphasized [71,84].

A reasonable conclusion from the totality of the data is that there is a role in improving adherence in higher-income countries, but specific nuances need to be considered on a case-by-case basis. There are limitations affecting the ability to make conclusions, including small sample sizes, heterogeneous populations, and heterogeneous interventions [53,54,60,62,80-82,88].

### Conclusions

This study attempted to review the existing literature on mHealth in HIV adherence, divided the literature to the largest subgroups for which evidence was found, and discussed existing studies with each of these groups as specific contexts.

On the basis of an evaluation of both the results and the quality of evidence available, we believe a clear role for mHealth interventions in developing countries exists; however, further work is needed before a final conclusion can be made in the higher-income countries, where the 2 main subgroup risk factors were MSM and IVDU. In addition, a gap in the literature in all 3 groups is the exact nature of interventions (optimal message frequency, content, and intervention themes).

In addition, as demonstrated by the variety and scope of interventions in our review, there is a difference between digital and mobile access, with some groups having the hardware and connectivity to access advanced apps such as smartphone apps, whereas other groups simply have mobile access and can only access text or SMS or voice call interventions.

Our review of the literature provides an optimistic outlook on the role of mHealth in improving HIV care; however, there are limitations to our work. Our narrative, compared with a systematic methodology, provides a broad and comprehensive overview of the subject area but does not have the stringent inclusion and exclusion criteria of a systematic review. However, our review provides a broad view of the subject and identifies specific focus areas for future systematic reviews. Specific areas of future work include better delineation of the efficacy of different types of interventions in higher-income countries and in specific risk groups within higher-income countries.

## Abbreviations

ARV: antiretroviral
IVDU: intravenous drug user
mHealth: mobile health
MSM: men who have sex with men
RCT: randomized controlled trial
