# Correction: mHealth Interventions to Promote Anti-Retroviral Adherence in HIV: Narrative Review

**DOI:** 10.2196/24250

**Published:** 2020-09-15

**Authors:** Stephen B Lee, Joanne Valerius

**Affiliations:** 1 Department of Medicine, Division of Infectious Diseases University of Saskatchewan College of Medicine Regina, SK Canada; 2 Department of Medical Informatics and Clinical Epidemiology Oregon Health and Science University Portland, OR United States

In “mHealth Interventions to Promote Anti-Retroviral Adherence in HIV: Narrative Review” (JMIR Mhealth Uhealth 2020;8(8):e14739) the authors noted one error.

In the originally published manuscript, 2nd author Joanne Valerius was noted as having contributed equally. This was incorrect and this note has now been removed.

The correction will appear in the online version of the paper on the JMIR Publications website on September 15, 2020, together with the publication of this correction notice. Because this was made after submission to PubMed, PubMed Central, and other full-text repositories, the corrected article has also been resubmitted to those repositories.

